# A systematic review on the appropriate discounting rates for the economic evaluation of gene therapies: whether a specific approach is justified to tackle the challenges?

**DOI:** 10.1017/S0266462324000096

**Published:** 2024-05-10

**Authors:** Tingting Qiu, Samuel Aballéa, Michal Pochopień, Mondher Toumi, Claude Dussart, Dan Yan

**Affiliations:** 1 Beijing Institute of Clinical Pharmacy, Beijing Friendship Hospital of Capital Medical University, Beijing, China; 2 Public Health Department, Aix-Marseille University, Marseille, France; 3 Faculté de Pharmacie, Université Claude Bernard Lyon 1, Lyon, France

**Keywords:** cost-effectiveness analysis, discount rate, gene therapies, health policy, reimbursement

## Abstract

**Objectives:**

Discounting the cost and effect for health intervention is a controversial topic over the last two decades. In particular, the cost-effectiveness of gene therapies is especially sensitive to the discount rate because of the substantial delay between the upfront cost incurred and long-lasing clinical benefits received. This study aims to investigate the influence of employing alternative discount rates on the incremental cost-effectiveness ratio (ICER) of gene therapies.

**Methods:**

A systematic review was conducted to include health economic evaluations of gene therapies that were published until April 2023.

**Results:**

Sensitivity or scenario analysis indicated that discount rate represented one of the most influential factors for the ICERs of gene therapies. Discount rate for cost and benefit was positively correlated with the cost-effectiveness of gene therapies, that is, a lower discount rate significantly improves the ICERs. The alternative discount rate employed in some cases could be powerful to alter the conclusion on whether gene therapies are cost-effective and acceptable for reimbursement.

**Conclusions:**

Although discount rate will have substantial influence on the ICERs of gene therapies, there lacks solid evidence to justify a different discounting rule for gene therapies. However, it is proposed that the discount rate in the reference case should be updated to reflect the real-time preference, which in turn will affect the ICERs and reimbursement of gene therapies more profoundly than conventional therapies.

## Introduction

Discounting is an economic method to adjust the values of the costs and benefits occurred in different time periods, reflecting the individual’s time preference for current benefits over future benefits (1). It is a near-universal consensus in the guidelines on the economic evaluation for health technology, employing discount rate for cost and effect to convert the future value to the present value is the standard practice (2).

However, there are arguments amongst the global academic community around the controversies in terms of the determination of appropriate discount rate for individual country by considering the varying economic growths rates (3). In the worldwide, discount rate is generally computed with two approaches. First approach relies on the measurements of social time preference using Ramsey formular, implying the discount rate should be determined depending on the pure time preference *δ*, the catastrophic risk premium *L*, and the wealth effect (i.e., combination of the marginal utility of consumption *μ* and the expected growth in income *g*) (4). For example, a discount rate of 3.5 percent recommended in the National Institute of Health and Care Excellence (NICE) guideline is determined by considering a time preference of 0.5 percent, a catastrophic risk premium of 1 percent, and a wealth effect value of 2 percent in the England (5). The second approach assumed that the discount rate should be estimated by the real return rate of a riskless investment, as approximated by the government bonds. Canadian Agency for Drugs and Technology in Health (CADTH) adopted this approach to decide a 1.5 percent discount rate by calculating the weighted average of the real provincial bond rates (6). Across the global, discount rate of 3 percent and 5 percent is most frequently recommended in the guidelines for health economic evaluation (3). However, it deserves mentioning that the justifications on the approaches of deciding discount rate are absent in most guidelines. This prevailed discount rate of 3 percent and 5 percent used might largely be influenced the early works done by the leading experts in the field of health economics (7). However, without adjusting the discount rate according to the economic conditions of individual countries, inappropriate discount rate may be used and potentially adversely affect the cost-effectiveness analysis (2).

Another critical controversy is around whether equal or differential discount rate should be applied for cost and effect (8;9). Outcome was discounted at the same rate as cost (i.e., equal discount) in almost all national guidelines for economic evaluation, with exceptions of the Netherland, Belgium, and Poland, where a lower discount rate for effect than cost was applied (1). The Netherlands recommends a discount rate of 4 percent for cost and 1.5 percent for benefit in order to take account of the increase in the value of health gains over time (10). Belgium recommends a discount rate of 3 percent for cost and 1.5 percent for benefit in order to avoid a too strong penalization of interventions that generate most of their benefits in the future (e.g., screening and vaccination programs) (1). Poland recommends a discount rate of 5 percent for cost and 3.5 percent for benefit, without providing justifications on this approach. The recommendation of equal discount rate is primarily based on the two influential arguments: consistency argument and postponing paradox. However, there is a long-lasting debate on whether nonmonetary outcome, such as QALYs, should be discounted differently as cost (differential discount). Criticism against both ‘consistency argument” and “postponing paradox” was raised in consideration of their limited relevance in the real-life decision-making process: the first statement neglected the possibility that the monetary value of health benefits such as QALYs will change over time, while the second statement is less critical for decision-makers who are confronting the problem of choosing between program A and existing program B, rather than choosing to recommend program A now or later (11;12).

Other debates are mainly related to whether discount rate should stay constant throughout the lifetime of the project (13); and whether non-reference discount rate is justified in special circumstance (1;14). As a result, the lack of agreements on the optimal analytic approach for discount rate raised methodological uncertainties in economic analysis (15). In particular, in the case of interventions that were associated with significant delay between the cost incurred and clinical benefits obtained, such as for vaccines, their cost-effectiveness will be profoundly influenced by the discount rate used (16).

One particular case is gene therapies, which are perceived as ground-breaking therapies that provide new promises for severe debilitating diseases with limited or no effective treatment options. However, there are substantial limitations in the clinical evidence of gene therapies, such as the small sample size and single-arm design, which resulted in significant challenges in collecting reliable input data for economic evaluation (17;18). One particular issue for the economic evaluation of gene therapies is whether discount rates recommended in methodological guidelines are appropriate, considering that gene therapies are generally associated with high one-time cost and potentially lifelong benefits (19;20). Although the cost-effectiveness analyses for gene therapies have received considerable attentions over the past 5 years, the issue of discount rate was rarely discussed. In this article, we aimed to highlight some of the ongoing debates on the methods for discount rate in health economic evaluation, and more importantly, we intended to comprehensively depict how the choice of discount rate will impact the cost-effectiveness results of gene therapies. Additionally, we discussed whether a specific discounting rule should be applied to gene therapies due to their distinct characteristic.

## Methods

This study was organized as follows: at first, we conducted a systematic review on the discount rates used in economic evaluations of gene therapies to investigate how the incremental cost-effectiveness ratios (ICERs) for gene therapies was changed according to the different discount rates used in the sensitivity analysis or scenario analysis. The systematic review is followed by a search of economic evaluation reports for gene therapies that have been released by England NICE until April 2023, aiming to understand whether NICE adopted a specific approach for choosing discount rate for gene therapies. Next, we presented a hypothetical example to illustrate how ICER values of gene therapies will change in case of using varying discount rate for cost and effect.

### Search strategy and inclusion criteria

**Figure 1.**
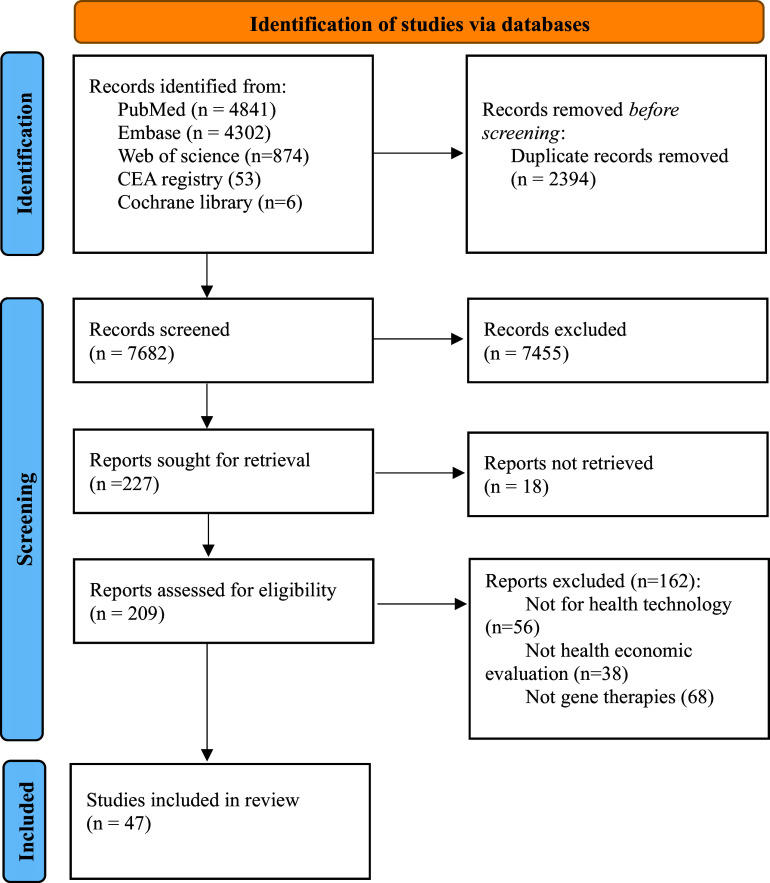
Preferred reporting items for systematic reviews and meta-analyses (PRISMA) diagram for the literature selection.

The systematic review was conducted in accordance with the Preferred Reporting Items for Systematic Reviews and Meta-Analyses (PRISMA) guideline. No protocol was developed and registered for this systematic review considering that no synthesized analyses were performed and our results were less likely to be biased. PubMed, Embase, Web of Science, Cochrane Library, and Cost-effectiveness Analysis Registry database were searched to identify the manuscripts published until April 2023 that satisfied the eligibility criteria: (i) the study type is cost-effectiveness, cost–benefit, or cost–utility analysis; (ii) the intervention to be examined is gene therapies including gene replacement therapies, gene editing therapies or chimeric antigen receptor (CAR) T cell therapies. Budget impact analyses as well as the economic evaluations for antisense oligonucleotides were excluded. CADTH search filters (21) for Economic Evaluations & Models for PubMed and Embase were applied. The language of publications was restricted to English, while no restriction on the scope of countries was applied. The judgments on whether the investigated products were gene therapies were made according to the definition of “gene therapy medicines” specified in the EU Regulation (EC) No 1394/2007. The PRISMA 2020 diagram (22) for the literatures search and selection was provided in the Figure 1. The detailed search strategy for five electronic databases was provided in the Supplementary Table S1.

### Data extraction and quality assessment

Data extraction was conducted in a pilot-tested Microsoft Excel template with information related to the study characteristic, method, results and conclusions was extracted (Supplementary Table S2). The quality of included economic evaluations was assessed with Drummond checklist (23), which is a well-known economic tool that considers: (i) the research question; (ii) the description of the study/intervention; (iii) the study design; (iv) the identification; (v) measurement; (vi) valuation of costs and consequences; (vii) discounting; (viii) incremental analysis; (ix) uncertainty of results and sensitivity analyses; and (x) discussion of the policy relevance and comparisons with existing literatures. For economic studies with discount rate considered in the sensitivity analyses, we calculated the change of ICERs comparing to base-case values in percentage form. The results were generally presented in a descriptive manner; no data synthesis of included studies was performed.

Two analysts independently screened the titles and abstracts (TTQ and SA), reviewed the full text (TTQ and MP), assessed the inclusions or exclusions of potentially eligible studies (TTQ and MP), and evaluated the quality of included studies (TTQ and SA). Any disagreements during the study selection, screening, and data extraction were resolved by discussion and consensus in the presence of senior authors (MT, CD, and DY) with expertise in health economics.

## Results

### Discount rate in the literatures on cost-effectiveness of gene therapies

A total of 47 studies investigating the cost-effectiveness or cost–utility of gene therapies were included after reading the full article for eligibility assessment (Figure 1). All included economic evaluations were rated of average or good quality (Supplementary Table S3). Most of the studies were conducted In the United States (N = 28), followed by the United Kingdom (N = 6) and the Netherlands (N = 3). In the base case analysis, all studies adopted discount rates for cost and effect that were recommended by the economic evaluation guideline of individual country. Specifically, discount rate of 4 percent was applied in the cost-effectiveness studies conducted in the Ireland; discount rate of 3.5 percent in the United Kingdom and Switzerland; discount rate of 3 percent In the United States, Germany, Spain, Singapore, and Sweden; discount rate of 2 percent in Japan; and discount rate of 1.5 percent in Canada. Equal discount rates for cost and effect were used in all studies, except for the three studies conducted in the Netherlands that discounted costs at 4 percent and discounted effects at 1.5 percent, which was in accordance with the Dutch national guidelines.

Among the included studies, nine studies did not consider the alternative discount rate, and 14 studies provided no information on whether discount rates were considered in sensitivity analysis or scenario analysis. The most common discount rate used in the sensitivity analyses or scenario analyses were 0 percent (i.e., no discounting applied), 1.5 percent (i.e., half of the common discount rate 3 percent), and 6 percent (i.e., twice of common discount rate 3 percent).

- **Alternative discounting with equal rate for cost and effect**

**Figure 2.**
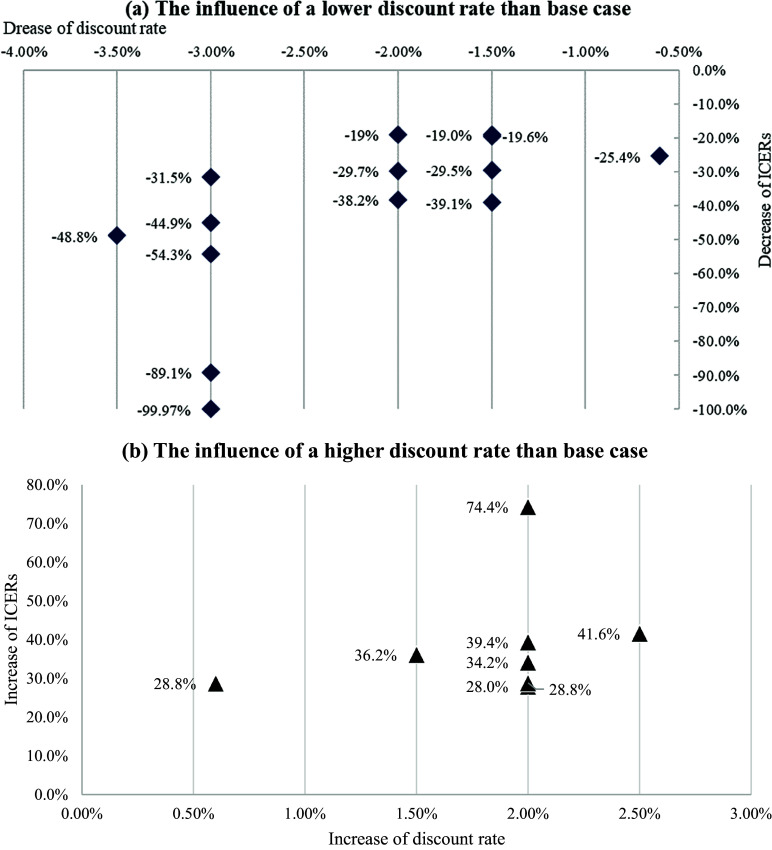
The influence of discount rate on the incremental cost-effectiveness ratio (ICER) estimates in the economic literatures.

Seven studies (24–30) provided graphs or figures (i.e., tornado diagram) to visualize the impacts of alternative discount rate on the cost-effective estimates, but they reported no detailed results on the magnitudes of ICERs changes compared to base-case analyses. Despite the absence of detailed ICERs results, all of them observed that ICERs of gene therapies were sensitive to the discount rate employed. For the remaining 15 studies (31–45), detailed ICERs results showing magnitude of impacts of discount rate were provided (Table 1, Figure 2). All studies (with the exception of the study by Liu et al. (33)) suggested that ICERs were positively related to discount rate, that is, ICERs of gene therapies versus comparators decreased when a lower discount rate was used, and vice versa. For the one exceptional study, different to the other studies comparing gene therapies with SoC or salvage therapy, Liu et al. (33) studied the ICERs of two CAR-T cell therapies, Yescarta® versus Kymriah®, for the treatment of diffuse large B-cell lymphoma (DLBCL) In the United States. It suggested that Yescarta® dominated Kymriah® in base case, while a lower discount rate slightly increased the ICERs of Yescarta® to $1,274/QALY.

- **Alternative discounting with differential rate for cost and effect**

**Table 1.** The impacts of discount rate on the cost-effectiveness estimates in the sensitivity or scenario analysis

References	Country	Gene therapies	Control	Disease	Discount rate in base case	Currency	ICER in base case	Discount rate in the sensitivity or scenario analysis	ICER in sensitivity or scenario analysis (percentage of change)
Broekhoff et al. (31)	Netherland	Zolgensma®	Spinraza and BSC	SMA I	4% for cost, 1.5% for benefit	EURO, €	138,875	3.5% for cost and effect	242,169 (74%)
								0% and 8% for cost	NA (NA)
								0% and 4% for effect	NA (NA)
Kansal et al. (32)	US	Zynteglo®	SoC	Transfusion–dependent β–thalassemia	3	USD, $	34,833	1.5% for cost and effect	Dominant (NA)
Liu et al. (33)	US	Yescarta®	Kymriah	Relapsed or refractory DLBCL	3	USD, $	Dominant	0% for cost and effect	$1,274 (NA)
							Dominant	6% for cost and effect	Dominant (NA)
Qi et al. (36)	US	Kymriah®	Salvage therapy	Relapsed or refractory DLBCL	3	USD, $	78,652	1.5% for cost and effect	63,450 (−19%)
								5% for cost and effect	100,850 (28%)
Salcedo et al. (34)	US	Hypothetical cell or gene therapy	SoC	Sickle cell disease	3	USD, $	140,877	1) Decrease 20%	105,118 (−25%)
								2) Increase 20%	181,407 (29%)
								0% for both cost and effect	15,332 (−89%)
Wakase et al. (35)	Japan	Kymriah®	SoC	Relapsed or refractory DLBCL	2	YEN (¥)	5,476,496	4% for cost and effect	7,633,458 (39%)
Jill 2020	Canada	Kymriah®	SoC	Patients with ALL	1.5	Canadian dollars $	141,000	3% for cost and effect	192,000 (36%)
Ribera Santasusana et al. (38)	Spain	Kymriah®	Salvage therapy	Relapsed and refractory ALL	3	EURO, €	28,819	0% for cost and effect	15,872 (−45%)
								5% for cost and effect	38,678 (34%)
Uhrmann et al. (39)	Germany	Luxturna®	SoC	Inherited retinal dystrophies	3	EURO, €	156,853	0% for cost and effect	Dominant (NA)
South et al. (40)	UK	Strimvelis®	HSCT	Adenosine deaminase deficiency–severe combined immune deficiency	3.5	GBP, £	120,506	1.5% for cost and effect	74,430 (−38%)
Malone et al. (41)	US	Zolgensma®	Spinraza and BSC	SMA1 and two copies of SMN2, before the age of 6 months	3	USD, $	31,379	0% for cost and effect; base case	14,347 (−54%)
								1.5% for cost and effect; base case	22,111 (−30%)
							57,261	0% for cost and effect; optimistic scenario	18.864 (−99.98%)
								1.5% for cost and effect; optimistic scenario	34,873 (−39%)
Roth et al. (42)	US	Yescarta®	Salvage therapy	Relapsed or refractory DLBCL	3	USD, $	58,146	0% for cost and effect	39,804 (−32%)
								5% for cost and effect	74,918 (29%)
Whittington et al. (43)	US	Kymriah®	Salvage therapy	Relapsed or Refractory Leukemia	3	USD, $	46,000	1.5% for cost and effect	37,000 (−20%)
Hettle et al. (44)	UK	CAR–T cell therapy	Salvage therapy		3.5	GBP, £	49,995	0% for cost and effect	25,576 (−49%)
								1.5% for cost and effect	35,162 (−30%)
								6% for cost and effect	70,808 (42%)
								0% for cost, 6% for effect	70,895 (42%)
								6% for cost, 0% for effect	25,544 (−49%)
Cher et al. (46)	Singapore	Kymriah®	Salvage therapy	DLBCL	3	SGD, $	508,531	0% for effect	440,114 (−13%)
								5% for effect	554,079 (9%)
								0% for cost	507,533 (−0.2%)
								5% for cost	510,392 (0.37%)
Petersohn et al. (45)	England	KTE–X19 CAR T therapy	SoC	Relapsed/refractory mantle cell lymphoma	3.5	GBP, £	67,713	1.5% for cost and effect	54,713 (−19%)
Carey et al. (47)	Ireland	Kymriah®	SoC	Relapsed/refractory ALL	4	EURO, €	73,086 73,086	1.5% for cost and effect 4% for costs, 1.5% for effect	55,630 (−31%) 50,260 (−45%)

ALL, acute lymphoblastic leukemia; BSC, best standard care; CAR-T, chimeric antigen receptor T cells; DLBCL, diffuse large B-cell lymphoma; HSCT, hematopoietic stem cell transplant; NA, not available; SMA, spinal muscular atrophy; SMN, survival of motor neuron; SoC, standard of care.

Broekhoff et al. (31) investigated that ICERs of Zolgensma® versus SoC for spinal muscular atrophy (SMA) type I in the Netherlands was most sensitive to discount rate for cost, followed by the discount rate for effect. Cher et al. (46) investigated that, compared to ICERs of S$508,531/QALY in the base-case using discount rate of 3 percent for effect, the ICERs of Kymriah® versus salvage chemotherapy for DLBCL in Singapore decreased to S$440,114/QALY (13 percent drop compared to base-case value) and increased to S$554,079/QALY (9 percent increase compared to base-case value) when a discount rate of 0 percent and 5 percent for effect was applied, respectively. The ICERs estimates were less sensitive to the discount rate for cost, with marginal changes in ICERs being observed when discount rate of 0 percent (S$507,533/QALY, 0.2 percent drop compared to base-case value) or 5 percent (S$510,392/QALY, 0.37 percent increase compared to base-case value) was used. Hettle et al. (44) applied a discount rate of 3.5 percent in base-case, and sensitivity analysis suggested that ICERs were less sensitive to discount rate for cost than discount rate for effect, with a 42 percent increase in ICERs (£49,995/QALY to £70,895/QALY, 42 percent increase compared to base-case value) of CAR-T cell therapy versus SoC when discount rate for cost and effect was 0 percent and 6 percent, respectively, and a 49 percent decrease in ICERs (£49,995/QALY to £25,544/QALY, 49 percent drop compared to base-case value) when discount rate for cost and effect was 6 percent and 0 percent, respectively. Carey et al. (47) applied a discount rate of 4 percent for cost and effect in base-case, and investigated that the baseline ICER (€73,086/QALY) was decreased to €55,630/QALY (31 percent drop compared to base-case value) when the discount rate was reduced to 1.5 percent for both cost and effect, and was further decreased to €50,260/QALY (45 percent drop compared to base-case value) when the discount rate was reduced to 1.5 percent only for effect. Cummings et al. (29) suggested that the ICERs were less sensitive to discount rate for cost than effect, as reflected in the sensitivity analyses showing the discount rate for effect was the most influential input with a larger impact on the ICER than the discount rate for cost.

### Discount rate used in the NICE appraisal reports for gene therapies

Eight gene therapies were evaluated by NICE until April 2023. The reference case discount rate of 3.5 percent was applied for all products, except for Zolgensma®, for which a non-reference discount rate of 1.5 percent was applied. Uncertainties in the maintenance of long-term benefits constituted the major reason for rejecting the non-reference case discount rate. Moreover, NICE was skeptical about whether people receiving Strimvelis® and Luxturna® would be considered to have “normal or near-normal health,” thus they claimed that both discount rates of 3.5 percent and 1.5 percent were taken into consideration. Additionally, NICE were also concerned that Libmeldy® as a single treatment will incur irrecoverable costs to National Health Service (NHS), combined with the ongoing costs of downstream treatments. Despite the significant uncertainties in the maintenance of long-term benefits and the debates around the capacity to achieve normal or near-normal health were also present for Zolgensma®, NICE decided a 1.5 percent discount rate was appropriate because of its potential for substantial long-term gains that may enable a higher quality of life for patients with SMA I and certain subgroup patients of SMA II. Advocacy from patient groups and clinical experts on the potential clinical benefits brought by Zolgensma® seems to greatly promote the application of 1.5 percent discount rate, stating that even if independent walking is not achieved in patients receiving Zolgensma® treatment, people who can sit independently can have a higher quality of life. The detailed information on the NICE evaluation of gene therapies is presented in Table 2.

**Table 2.** NICE evaluation for the gene therapies

Products	Discount rate in base case (%)	Base case ICER per QALY	1.5% discount rate is considered or not	ICER per QALY with 1.5% discount rate	Decision for recommendation
Luxturna®	3.5	£114,956–£155,750, depending on the use of utility values	–Highly uncertainty about 1) whether people who had Luxturna would be considered to have “normal or near–normal health” and 2) whether the long–term benefits of treatment would be achieved because of the limited evidence. –Both discount rates during its decision–making. However, it preferred the use of 3.5% because it was uncertain about whether Luxturna fully met the criteria for using a discount rate of 1.5%.	£60,908 and £86,118 depending on the use of utility values	–Recommended with commercial agreements. –HST with a QALY weight of 1.2.
Zolgensma®	1.5	Not given	Despite these uncertainties, the committee concluded that Zolgensma meets the criteria for using a 1.5% discount rate and this would be used for decision–making.	Not given	–Recommended with commercial agreements (for SMA I) or managed access agreement (for SMA II) –HST with a QALY weight of lower than 1.86.
Strimvelis®	3.5	£91,910 and £84,172 gained compared with an HSCT from a MUD and a haploidentical donor	–Highly uncertainty about (i) whether people who had Luxturna would be considered to have “normal or near–normal health” and (ii) whether the long–term benefits of treatment would be achieved because of the limited evidence. –Both discount rates during its decision–making. However, it preferred the use of 3.5% because it was uncertain about whether Strimvelis® fully met the criteria for using a discount rate of 1.5%.	£36,360 and £14,645 per QALY gained compared with an HSCT from a MUD and a haploidentical donor respectively	–Recommended – HST with a QALY weight of 1.40 and 1.96 for Strimvelis® compared with an HSCT from a MUD and a haploidentical donor respectively
Libmeldy®	3.5	Not given	There was substantial uncertainty about how long benefits of OTL–200 last. It noted that OTL–200’s cost is a single cost that could commit the NHS to significant irrecoverable costs. And there are also potential ongoing irrecoverable costs for patients who have OTL–200 and stabilize in worse health states for longer periods. So, the committee considered that the non–reference discount rate of 1.5% was not appropriate for decision–making	Not given	–Recommended with commercial agreements –HST with a QALY weight of 1–3. But the exact weighting was uncertain and dependent on the MLD subgroup
Tecartus®	3.5	£58,223	Not given	Not given	CDF with managed access agreement
Yescarta®	3.5	Over £50,000	The committee noted that Yescarta appeared clinically effective, but was aware that the evidence was immature so the duration of health benefits could not robustly show cure	Not given	CDF with managed access agreement
Kymriah®	3.5	£55,403 gained with a 4–year cure point	Not given	Not given	CDF with managed access agreement
Imlygic®	3.5	£23,900 and £24,100 compared to dacarbazine and best supportive care, retrospectively	Not given	Not given	Recommended with commercial agreements.

CDF, cancer drug fund; HS, highly specialized technology; HSCT, hematopoietic stem cell transplant; ICER, incremental cost-effectiveness ratio; QALY, quality-adjusted life year; SMA, spinal muscular atrophy.

### Impact of the discount rate: a hypothetical example

**Figure 3.**
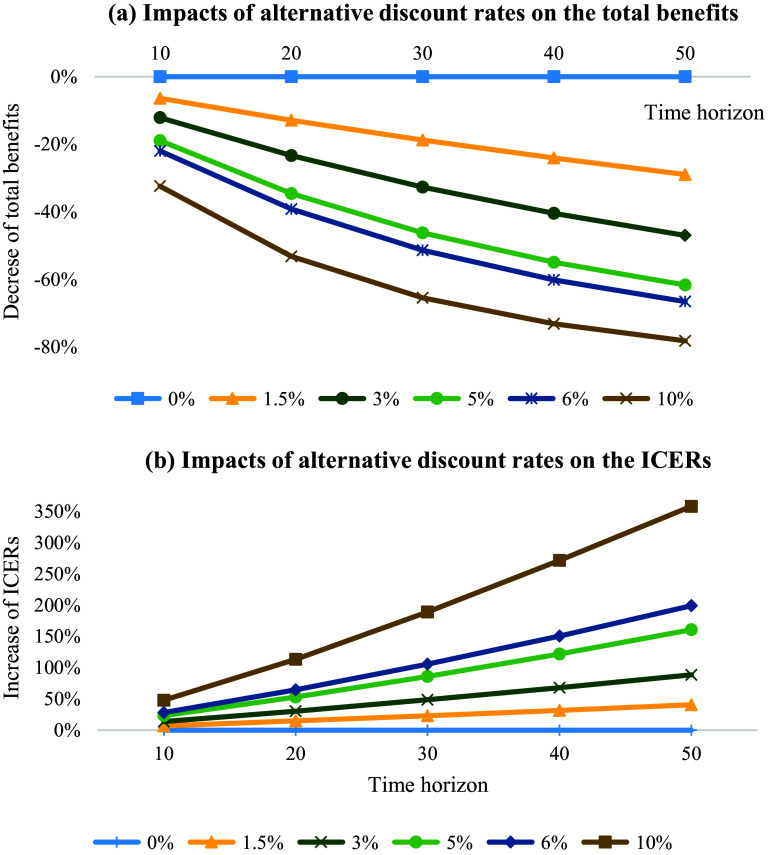
Impacts of differing discount rate on the total benefits over time.

Gene therapies are generally associated with high upfront cost and substantial long-term benefits, implying that discount rate will have significant impacts on their cost-effectiveness results (44;48;49). For instance, assuming a hypothetic gene therapy costs $1million for one-time administration for one person, and it constantly generates one unit of benefit annually. As showed in the Figure 3, compared to no discount rate, using 5 percent discount rate will result in an 18.92 percent, 34.57 percent, 46.20 percent, 54.96 percent, 61.66 percent decrease in the total benefits gained for the time horizon of 10 years, 20 years, 30 years, 40 years, and 50 years, respectively. The difference in the total benefits gained will become larger with the increase of discount rate used and the prolongation of time horizon. Considering the one-time treatment costs for gene therapies that are incurred at the initiation of project will not be discounted, the substantial change in the valuation of health benefits will significantly affect the cost-effectiveness estimates. In general, a lower discount rate will result in more favorable ICERs for gene therapies (50). As showed in the Figure 3, compared to no discount rate, using 5 percent discount rate will result in a 23.34 percent, 52.84 percent, 85.86 percent, 122.01 percent, 160.84 percent increase in the ICERs for the time horizon of 10 years, 20 years, 30 years, 40 years, and 50 years, respectively (Figure 3).

## Discussion

### The impacts of discount rate on the cost-effectiveness of gene therapies

Due to distinct characteristics of gene therapies, such as the high upfront cost and potentially long-term benefits, their cost-effectiveness outcomes are particularly sensitive to the discount rate. As noted in this review, varying the discount rate, especially for health effect, could be powerful to alter the conclusion on whether gene therapies are cost-effective, thus influencing the final decisions of reimbursement. For example, in the study by Salcedo et al. (34), increasing a discount rate of 3 percent by 20 percent in the cost-effectiveness analysis of gene therapy for the treatment of sickle cell disease In the United States increased the ICERs to $181,407, which exceeded the threshold of $100,000–$150,000 informally specified by the US health economic guideline (51). Similarly, Wakase et al. (35) investigated that there was only one scenario made the ICERs of Kymriah® exceeded the threshold of ¥7,500,000 ($51,555,00) in Japan, that is, when increasing discount rate from 2 percent to 4 percent.

### The appropriateness of discount rate in reference case

Before embarking on the debates on the appropriate discount rate for gene therapies, one paramount question is whether the reference case discount rate for conventional medicines recommended in the health economic guidelines is reasonable. For example, the discount rate of 3.5 percent adopted by NICE is criticized to be too high because there is only weak evidence supporting the parameters used for Ramsey equation (52). Specifically, although it is feasible to derive the elasticity and the growth rate of consumption from the existing data, it is challenging to accurately estimate the pure time preference and catastrophic risk parameter (9;53;54). Moreover, there is a fundamental question on whether the utilization of Ramsey formula to determine the discount rate is appropriate in the UK setting (55). One consensus paper by Claxton and Paulden et al. (56) implied that the discount rate should be determined depending on the decision-makers’ perspective and whether they are operating under a constrained budget. For a decision maker that takes *social decision-making* perspective and operates under a *constrained* budget, whose responsibility is to maximize the value of population health, the appropriate discount rate should be determined by real interest rate faced by the higher authority that funds the health system (52). This could be approximated by the real borrowing cost faced by the government, as reflected in the CADTH guideline (52;55). Therefore, it is suggested that government borrowing cost, rather than the Ramsey formula, is more relevant to compute the discount rate in the United States. However, the discount rate of 3.5 percent currently adopted is substantially higher than the real borrowing cost (less than 1 percent as of April 2023) faced by the UK government (55).

In the United States, the discount rate of 3 percent for cost and benefit was adopted considering that the discount rate should reflect the return of a riskless, long-term investment (3). However, Paulden et al. (54) challenged this statement given that official estimates of the real rate of return on US government bonds were approximately 0.3–1.5 percent annually, thus discount rate ≤1.5 percent for costs and health effects seemed theoretically and empirically defensible. Likewise, Devlin et al. (57) were concerned that the discount rate of 5 percent recommended in the Australia was too high compared to other counterpart developed countries. They implied that, compared to a lower discount rate, a higher discount rate would increase the cost-effectiveness and reduce the possibility of reimbursement of the gene therapies that potentially offer lifetime benefits to life-threatening diseases (57). Therefore, more evidence on the appropriate reference case discount rate for conventional medicines is warranted prior to the conversations on whether different methods for discount rates are justified for in specific situations, such as for gene therapies.

### Equal or differential discount rate for gene therapies

Considering that most of the included studies varied the discount rate for cost and effect to the same rate in the scenario analyses, limited evidence was available to investigate the impacts of differential discount rate for cost and effects. The study by Hettle et al. (44) suggested that ICERs of investigated CAR-T cell therapies were more sensitive to the discount rate for effect than discount rate for cost. The small impact of discount rate for cost on ICERs is explained by the fact that the price of CAR-T cell therapy (£358,057) represented the largest proportion of the total cost (£449,128), and it was not discounted because the CAR-T was one-time treatment only administrated in the first year. This explicitly suggests that, irrespective of the discount rate for costs, a lower discount rate for effect could have a significant effect on the ICERs result and reimbursement decisions of gene therapies.

However, in case of healthcare system with constrained budgets, the differential discount rate for cost and effect will be justified only if the cost-effectiveness threshold is adjusted over time (56). Despite that a lower discount rate for effect will favor the cost-effectiveness results and reimbursement decisions, the adoption of differential discount rate should not be simply driven by the motivation of accelerating the patient access to innovative gene therapies. Instead, equal discount rate, as most of the health economic guidelines recommended, will remain the mainstay until more studies investigating the trend for the change of cost-effectiveness threshold is available to justify a differential discount rate (58).

### Nonconstant discount rate for gene therapies

Another critical issue is around the choice between constant discount rate and nonconstant discount rate. The endorsement of the nonconstant discount rate was supported by the observations of decreasing timing aversion for cost and effect in several time preference studies (13;59;60). The use of nonconstant discount rate appears relevant for gene therapies because they potentially offer lifelong benefits for patients with disease onset in the early childhood. Also, time horizon of lifetime is commonly considered in the cost-effectiveness of gene therapies. Among the included studies, study by Hettle et al. (44) were the only study that examined the impacts of step discounting of 3.5 percent up to year 30, 3 percent thereafter for both costs and health effects, which was in accordance with the recommendations from the UK Treasury. They observed only a negligible difference between ICERs based on a stepwise discount rate and ICER based on a constant discount rate (£49,601/QALY vs. £49,995/QALY). Although the explanation for this finding is not provided, it seems less surprising because of the small change of discount rate employed. Moreover, it is questionable if the difference of 0.5 percent in discount rate could adequately represent the real-time preferences of general public. For example, one Dutch study by Parouty et al. (61) estimated that the average annual discount rate for benefits declined from 5 percent in 5 years to 1.7 percent in 20 years and 0.8 percent in 40 years, respectively. This implies that a greater magnitude of decrease in the discount rate than 0.5 percent decrease as recommended by UK Treasury could be more reflective of the real practice and more valuable to provide new evidence on the impacts of nonconstant discount rates.

### Non-reference case discount rate for gene therapies

In most countries, one universal discount rate was recommended in methodological guidelines for economic evaluation irrespective of the type of healthcare intervention. However, England NICE suggested a non-reference case discount rate of 1.5 percent for both cost and effect when all the following criteria were satisfied: (i) the technology is indicated for life-threatening diseases, (ii) it is likely to restore them to full or near-full health, (iii) the benefits are likely to be sustained over a very long period; and at last, (iv) the intervention will not commit the NHS to significant irrecoverable costs (62;63).

However, the eligibility criteria of non-reference case were criticized to be ambiguous and unjustified (58), such as how to define the “sustained benefits over a long period” and “full or near-full health.” Moreover, this raises another critical question of whether this non-reference case discount rate would be applicable for gene therapies. In a “mock technology appraisal” report, Hettle et al. (44) indicated that non-reference case of 1.5 percent discount rate was inappropriate for CAR-T cell therapy, considering that its application could generate significant debates in future appraisals due to the lack of precedents. Moreover, they recognized that it remained unclear on the sustainability of long-term benefits of regenerative medicine and potentially irrecoverable cost for NHS. Marsden and Towse (64) responded to the report by Hettle et al. (44) and commented that the requirement of restricting the 1.5 percent discount rate to technologies that “do not incur significant irrecoverable costs” was arbitrary and irrelevant, because this requirement seems not be based on the consideration for efficiency, but rather to limit the number of technology that will be eligible for a lower discount rate.

Although non-reference case discount rate in NICE will improve the ICER results and promote the reimbursement of gene therapies that would otherwise be rejected, it appears not straightforward to assess whether the predefined criteria will be satisfied (58). For example, despite the obvious evidence limitations similar as other gene therapies, non-reference case discount rate of 1.5 percent was applied to Zolgensma® but not others. More clarifications and consistencies in the definition of each criterion, such as “full or near-full health,” “over a very long period” and “significant irrecoverable cost,” is paramount to increase the transparency in the use of non-reference case of discount rate in special circumstance.

More importantly, the justifications for utilizing a different discount rate for gene therapies should be examined in the empirical studies investigating whether the social time preference varies between conventional therapies offering incremental benefits for less severe diseases and life-changing therapies offering potentially lifetime benefits for severe diseases. Without such evidence, applying a different discount rate for gene therapies is baseless and likely unfairly favors gene therapies over other innovative therapies.

### Arguments from literatures on the discount rate for gene therapies

The significance of the discount rates on economic evaluations of gene therapies has stimulated discussions on whether the existing reference case is appropriate and whether a specific discount rate is justified for gene therapies. In general, published opinions do not support a different discount rate for gene therapies. Vellekoop et al. (65) pointed out that a different discount rate for gene therapy will hamper the comparability of cost-effectiveness across interventions considering that other conventional therapies may also provide broader societal and long-term benefits. Likewise, Drummond et al. (50) elaborated that there was no strong evidence supporting that a different method for discount rate should be applied for gene therapy, but they recommended to explore the impacts of different discount rate used on the cost-effectiveness results. Moreover, Jönsson et al. (66) recommended to establish an international, multi-disciplinary forum to consider the economic, social, and ethical implications of the choice of the differential or equal discount rate for cost and effect in a variety of circumstances. The summary of evidences regarding whether gene therapies merit a specific discount rate was provided in the Supplementary Table S4.

### Limitation of this study

To the best of our knowledge, this is the first systematic review that summarizes the issues of discount rate adopted in the economic evaluation of gene therapies. Despite the valuable evidence generated from this study, some limitations existed. First, multiple economic evaluations provided no details on the parameters included in the sensitivity analysis or scenario analysis, making it impossible to know whether the impact of discount rate was examined. Second, we have limited compacity to investigate to what extent the change of ICERs due to varying discount rate used will alter the reimbursement decisions given that the official ICER threshold is lacking in most countries. Third, the quality of economic analyses was evaluated with Drummond checklist, instead of the CHEERS 2022 checklist, which may cause overestimation of quality of the included studies. Finally, most of included studies were economic evaluations of CAR-T cell therapies conducted in developed countries, limiting the generalizability and applicability of our conclusion to the low-and-middle income countries.

### Implications for practice and policy

Although it is acknowledged that the cost-effectiveness of gene therapies is highly sensitive to the discount rate, and a lower discount rate will result in a more favorable reimbursement decision, it is noteworthy that there may already exist other evaluation pathways aiming to encourage the adoption and improve access of innovative gene therapies. For example, the highly specialized technologies (HST) program is implemented in NICE for very rare diseases that are severely disabling and have no satisfactory treatments available. Through HST program, a more generous cost-effectiveness threshold of £100,000–£300,000 (compared to £20,000–£30,000 for non-HST technologies) is allowed, depending on the number of QALYs added. Among the gene therapies assessed by NICE, four products, Strimvelis®, Luxturna®, Zolgensma®, and Libmeldy®, were recommended through HST pathway, combined with commercial agreements to be implemented. The Cancer Drug Fund (CDF) is another source of funding for promising oncology products providing important clinical benefits but showing significant limitations in clinical evidence. Three CAR-T cell therapies, Yescarta®, Kymriah®, and Tecartus®, were all accepted to be used within CDF. Therefore, gene therapies may have a higher likelihood of reimbursement due to their eligibilities for special evaluation pathways or funding programs. If this is the case, a separate rule of discount rate for gene therapies are likely to offer “double” benefits for them, causing potential inequity issues for getting access to conventional products targeting chronic and less severe diseases.

## Conclusion

The reference case discount rates currently recommended in the pharmacoeconomic guidelines (3) are not sufficiently justified and not necessarily relevant to the real social time preference. Due to the distinct characteristic of gene therapies, their cost-effectiveness results are highly sensitive to the discount rate, where the cost-effectiveness is improved in case that a decreased discount rate for effect is used. Consequently, inappropriate discount rate will substantially distort the cost-effectiveness of gene therapies in particular. With the predefined ICER threshold, the application of alternative discount rate could be influential to reverse the reimbursement decision. There are growing arguments advocating for the adjustment of discount rate in the reference case in general, which might have more profound impacts on the cost-effectiveness of gene therapies than other treatments. However, there is no strong evidence supporting a different discount rate for gene therapies than for other treatments. Moreover, given the constrained budgets, any adjustments on the discounting rule for gene therapies must also adequately take the financial affordability into account. More research on the social time preferences toward such innovative technologies compared with conventional treatments is needed to provide more answers on whether gene therapies should be entitled to a special discounting rule.

## Supporting information

Qiu et al. supplementary material Qiu et al. supplementary material

## Data Availability

Search strategies used for the systematic literature review and the selection criteria are available in the Supplementary files. The excel file used for the data extraction during the systematic literature review can be made available upon request.

## Supplementary material

The supplementary material for this article can be found at http://doi.org/10.1017/S0266462324000096.
