# Supplementary material for: A systematic review on the appropriate discounting rates for the economic evaluation of gene therapies: whether a specific approach is justified to tackle the challenges?
Source: Int J Technol Assess Health Care. 2024 May 10;40(1):e23. doi: 10.1017/S0266462324000096 (PMC11569993; doi:10.1017/S0266462324000096)
Supplement: Qiu et al. supplementary material [file S0266462324000096sup001.docx]

**Supplementary material**

**Supplementary Table 1** Search strategy for 5 databases

| **Database** | **Search strategy** | **Results** | **Date** |
| --- | --- | --- | --- |
| PubMed | ("Economics"[Mesh:NoExp] OR "Costs and Cost Analysis"[mh] OR "Economics, Nursing"[mh] OR "Economics, Medical"[mh] OR "Economics, Pharmaceutical"[mh] OR "Economics, Hospital"[mh] OR "Economics, Dental"[mh] OR "Fees and Charges"[mh] OR "Budgets"[mh] OR budget*[tiab] OR economic*[tiab] OR cost[tiab] OR costs[tiab] OR costly[tiab] OR costing[tiab] OR price[tiab] OR prices[tiab] OR pricing[tiab] OR pharmacoeconomic*[tiab] OR "pharmaco-economic*"[tiab] OR expenditure[tiab] OR expenditures[tiab] OR expense[tiab] OR expenses[tiab] OR financial[tiab] OR finance[tiab] OR finances[tiab] OR financed[tiab] OR "value for money"[tiab] OR "monetary value*"[tiab] OR "models, economic"[mh] OR "economic model*"[tiab] OR "markov chains"[mh] OR markov[tiab] OR "monte carlo method"[mh] OR "monte carlo"[tiab] OR "Decision Theory"[mh] OR "decision tree*"[tiab] OR "decision analy*"[tiab] OR "decision model*"[tiab]) AND (("gene treatment*"[Title/Abstract] OR "genetic treatment*"[Title/Abstract] OR "molecular therap*"[Title/Abstract] OR "antisense therap*"[Title/Abstract] OR "gene replacement therap*"[Title/Abstract] OR "genetic immunization*"[Title/Abstract] OR "oligonucleotide therap*"[Title/Abstract] OR "ribozyme therap*"[Title/Abstract] OR "RNAi therapeutic*"[Title/Abstract] OR "T-cell therapy"[Title/Abstract])) | 4841 | April 2023 |
| Embase | ("gene treatment*" or "genetic treatment*" or "molecular therap*" or "antisense therap*" or "gene replacement therap*" or "genetic immunization*" or "oligonucleotide therap*" or "ribozyme therap*" or "RNAi therapeutic*" or "T-cell therapy").ab,kw,ti. AND (Economics/ or Cost/ or exp Health Economics/ or Budget/ or budget*.ti,ab,kf. or (economic* or cost or costs or costly or costing or price or prices or pricing or pharmacoeconomic* or pharmaco-economic* or expenditure or expenditures or expense or expenses or financial or finance or finances or financed).ti,kf. or (economic* or cost or costs or costly or costing or price or prices or pricing or pharmacoeconomic* or pharmaco-economic* or expenditure or expenditures or expense or expenses or financial or finance or finances or financed).ab. /freq=2 or (cost* adj2 (effective* or utilit* or benefit* or minimi* or analy* or outcome or outcomes)).ab,kf. or (value adj2 (money or monetary)).ti,ab,kf. or Statistical Model/ or economic model*.ab,kf. or Probability/ or markov.ti,ab,kf. or monte carlo method/ or monte carlo.ti,ab,kf. or Decision Theory/ or Decision Tree/ or (decision* adj2 (tree* or analy* or model*)).ti,ab,kf. | 4302 | April 2023 |
| Cochrane Library | TI/AB/KW=("cost-effectiveness analyses" or "cost-utility analyses" or "cost-benefit analyses" or "economic evaluations" or "economic evaluation" or "Pharmacoeconomics" or "health economic" or "cost-effectiveness" or "cost effectiveness" or "cost-utility" or "cost utility" or "cost-benefit" or "cost benefit" or "health technology assessment") AND  TI/AB/KW= ("gene treatment*" or "genetic treatment*" or "molecular therap*" or "antisense therap*" or "gene replacement therap*" or "genetic immunization*" or "oligonucleotide therap*" or "ribozyme therap*" or "RNAi therapeutic*" or "T-cell therapy") | 6 | April 2023 |
| Web of science | TS=("Value of life" OR "value for money" OR "quality adjusted life year*" OR qaly* OR qald* OR qale* OR "disability adjusted life year*" OR daly*) OR TS=(economic* or cost or costs or costly or costing or pharmacoeconomic*) OR TS=(expenditure* OR "willingness to pay" OR budget*)  TS= ("gene treatment*" or "genetic treatment*" or "molecular therap*" or "antisense therap*" or "gene replacement therap*" or "genetic immunization*" or "oligonucleotide therap*" or "ribozyme therap*" or "RNAi therapeutic*" or "T-cell therapy") | 874 | April 2023 |
| CEA registry | Basic search with keywords ‘Gene therapy’ | 53 | April 2023 |

**Supplementary Table 2** Extracted items for Extraction form

| **Study characteristic** | **Method** | **Results and conclusion** |
| --- | --- | --- |
| Study ID | Software used | Result |
| Authors, Year | Type of model | Incremental cost |
| Country | Time horizon | Incremental QALY |
| Title | Cycle | Incremental LY |
| Journal | Perspective | ICER |
| Sponsor | Transition probability | Budget impact |
| Language | Key assumption | DSA analysis |
| Objective | Extrapolation | PSA analysis |
| Type of analysis | Efficacy outcome | Scenario analysis |
| Data source | Utility assessment | Limitations |
| Intervention | Discount rate | Conclusion |
| Comparator | Currency | Other comments |
| Patient character | Cost year |  |
| Patient number | ICER Threshold |  |
|  | Sensitivity analysis |  |

**Supplementary Table 3** Quality assessment of economic evaluations using the Drummond checklist

| **First author** | **Year** | **Q1** | **Q2** | **Q3** | **Q4** | **Q5** | **Q6** | **Q7** | **Q8** | **Q9** | **Q10** | **Quality*** |
| --- | --- | --- | --- | --- | --- | --- | --- | --- | --- | --- | --- | --- |
| Almutairi A.R. | 2019 | √ | √ | √ | × | √ | √ | × | √ | √ | √ | Good |
| Ball G. | 2022 | √ | √ | √ | × | √ | √ | × | √ | √ | √ | Good |
| Bolous N.S. | 2021 | √ | √ | √ | × | √ | √ | √ | √ | √ | √ | Good |
| Broekhoff T.F. | 2021 | √ | √ | × | √ | √ | √ | √ | √ | √ | √ | Good |
| Carey N | 2022 | √ | √ | √ | √ | √ | √ | √ | √ | √ | √ | Good |
| Cher B.P. | 2020 | √ | √ | √ | Unclear | √ | √ | √ | √ | √ | √ | Good |
| Choe J.H. | 2022 | √ | √ | √ | √ | √ | √ | √ | √ | √ | √ | Good |
| ConXck | 2020 | √ | √ | Unclear | Unclear | √ | √ | × | √ | × | × | Average |
| Cummings Joyner A.C. | 2018 | √ | √ | √ | √ | √ | √ | √ | √ | √ | √ | Good |
| Daniel V. | 2020 | √ | √ | √ | √ | √ | √ | √ | √ | √ | √ | Good |
| Dean R. | 2021 | √ | √ | √ | √ | √ | √ | √ | √ | √ | √ | Good |
| Halioua H. | 2019 | √ | √ | √ | × | × | × | √ | × | √ | √ | Average |
| Hettle R. | 2017 | √ | √ | √ | √ | √ | √ | √ | √ | √ | √ | Good |
| Jill F. | 2020 | √ | √ | √ | √ | √ | √ | √ | √ | √ | √ | Good |
| Johnson S. | 2019 | √ | √ | √ | √ | √ | √ | √ | √ | √ | √ | Good |
| Jorgensen J. | 2018 | √ | √ | × | Unclear | √ | √ | √ | × | √ | √ | Average |
| Kambhampati S-a | 2022 | √ | Unclear | √ | √ | √ | × | √ | √ | √ | √ | Good |
| Kambhampati S-b | 2022 | √ | √ | √ | √ | √ | × | √ | √ | √ | √ | Good |
| Kambhampati S-c | 2023 | √ | √ | √ | √ | √ | √ | √ | √ | √ | √ | Good |
| Kansal A.R. | 2021 | √ | √ | √ | Unclear | √ | √ | √ | √ | √ | √ | Good |
| Keziah C. | 2020 | √ | √ | Unclear | √ | √ | √ | √ | Unclear | X | √ | Average |
| Lin J.K. | 2019 | √ | √ | √ | Unclear | √ | √ | √ | √ | √ | √ | Good |
| Lin J.K. | 2018 | √ | √ | Unclear | Unclear | √ | √ | √ | √ | √ | √ | Good |
| Liu R. | 2021 | √ | √ | × | × | √ | √ | √ | √ | √ | √ | Good |
| Machin N. | 2018 | √ | √ | √ | × | √ | √ | √ | √ | √ | √ | Good |
| Malone D.C. | 2019 | √ | √ | √ | Unclear | √ | √ | √ | √ | √ | √ | Good |
| Moradi-Lakeh | 2021 | √ | √ | √ | Unclear | √ | √ | √ | √ | √ | √ | Good |
| Perales M.A. | 2022 | √ | √ | √ | √ | √ | √ | √ | √ | √ | √ | Good |
| Petersohn S. | 2022 | √ | √ | √ | √ | √ | √ | √ | √ | √ | √ | Good |
| Potnis K.C. | 2023 | √ | √ | √ | √ | √ | √ | √ | √ | √ | √ | Good |
| Qi C.Z. | 2021 | √ | √ | √ | Unclear | √ | √ | √ | √ | √ | √ | Good |
| Roth J.A. | 2018 | √ | √ | √ | Unclear | √ | √ | √ | √ | √ | √ | Good |
| Salcedo J. | 2021 | √ | √ | √ | Unclear | √ | √ | √ | √ | √ | X | Good |
| Santasusana J.M.R. | 2020 | √ | √ | √ | Unclear | √ | √ | √ | √ | √ | √ | Good |
| Sarkar R.R. | 2019 | √ | √ | √ | √ | √ | √ | √ | √ | √ | √ | Good |
| Shih S.T.F. | 2021 | √ | √ | √ | √ | √ | √ | √ | √ | √ | √ | Good |
| Simons C.L. | 2021 | √ | √ | √ | Unclear | √ | √ | √ | √ | √ | √ | Good |
| South E. | 2019 | √ | √ | √ | × | √ | √ | √ | × | √ | √ | Good |
| Ten Ham R | 2022 | √ | √ | √ | √ | √ | × | √ | √ | √ | √ | Good |
| Thielen F.W. | 2020 | √ | √ | √ | √ | √ | √ | √ | √ | √ | √ | Good |
| Uhrmann M.F. | 2020 | √ | √ | √ | √ | √ | √ | √ | Unclear | √ | √ | Good |
| Vijenthira A. | 2022 | √ | √ | √ | √ | √ | × | √ | √ | √ | √ | Good |
| Wakase S. | 2021 | √ | √ | √ | × | √ | √ | √ | √ | √ | √ | Good |
| Walton A. | 2019 | √ | √ | √ | Unclear | √ | Unclear | √ | × | √ | √ | Good |
| Whittington M.D. | 2018 | √ | √ | √ | Unclear | √ | √ | √ | √ | √ | √ | Good |
| Whittington M.D. | 2019 | √ | √ | √ | Unclear | √ | √ | √ | √ | × | √ | Good |
| Zimmermann M. | 2019 | √ | √ | √ | √ | √ | √ | √ | √ | √ | √ | Good |
| ZuluagaSanchez S. | 2019 | √ | √ | √ | √ | √ | √ | √ | √ | √ | √ | Good |

*poor quality (1–3 points), average quality (4–7 points) and good quality (8–10 points).

Q1: Research question well defined? Q2: Comprehensive description of alternatives? Q3: Effectiveness of program established? Q4: Important & relevant costs & consequences for each alternative identified? Q5: Costs & consequences measured accurately & appropriately? Q6: Costs & consequences valued credibly? Q7: Costs & consequences adjusted for differential timing? Q8: Incremental analysis of costs & consequences performed? Q9: Allowance made for uncertainty in estimates? Q10: Presentation & discussion of study results include all issues of concern to users?

**Supplementary Table 4** Summary of arguments on whether gene therapies merit a specific discounting rule

|  | **Justifications and advantages** | **Uncertainties and disadvantages** |
| --- | --- | --- |
| Non-reference case with a lower discount rate for gene therapies than conventional therapies | - Give priority to interventions satisfying the pre-defined criteria, normally the ones that target severe conditions with high unmet medical needs. - Improve the cost-effectiveness estimates of gene therapies, increase the possibility of being reimbursed. | - Uncertainties existed in whether gene therapies have the substantial clinical benefits maintaining over a long time, and whether gene therapies will incur uncoverable expense to the healthcare budget. - There lacks empirical evidence to illustrate whether the social rate of time preference is different for gene therapies compared to conventional therapies - Without clarifications on the eligible criteria, inconsistences will be shown in determining the appropriate discount rate, potentially leading to inequality in the decision making. |
| Differential discount rate with a lower discount rate for benefits than cost | - The two influential arguments supporting equal discount rate- consistency argument and postponing paradox is criticized for lack of relevance in real life. - Prevent the long-term benefits of gene therapies from being discounted too heavily, leading to the underestimation of the long-term benefits. | - As a consensus from leading experts, differential discount rate for cost and benefits was justified if either the consumption value of health (v) or the cost-effectiveness threshold (k) is changing over time. However, current evidence is lacking to enable the analysis on the change patterns of v or k - Empirical evidence with varying methodologies showed discrepant findings on whether social rate of time preference for cost is higher than benefit. |
| Non-constant discount rate over time for gene therapies | - Empirical evidence suggested that time preferences (discount rate) for cost and benefits declined over time - Prevent the long-term benefits of gene therapies from being discounted too heavily, leading to the underestimation of the long-term benefits | - Uncertainties existed in whether gene therapies have the substantial clinical benefits maintaining over a long time - It is debatable in terms of how to determine the cut-off time and define the appropriate discount rate that is decreasing over time. For example, non-constant discount rate will have marginal impact on the cost-effectiveness results if the cut-off time is set to be too far from the initial time because few studies will have time horizon long enough to be eligible for non-constant discount rate. |
